# 2-(2,4-Difluoro­phen­yl)-5-nitro­pyridine

**DOI:** 10.1107/S1600536812024713

**Published:** 2012-06-02

**Authors:** Feng Sun, Xuan Shen, Rui Zhao, Xin Wang, Dun-Ru Zhu

**Affiliations:** a State Key Laboratory of Materials-Oriented Chemical Engineering, College of Chemistry and Chemical Engineering, Nanjing University of Technology, Nanjing 210009, People’s Republic of China

## Abstract

In the title mol­ecule, C_11_H_6_F_2_N_2_O_2_, the benzene and pyridine rings form a dihedral angle of 32.57 (6)°. The nitro group is tilted with respect to the pyridine ring by 12.26 (9)°. An intra­molecular C—H⋯F hydrogen bond is present. In the crystal, mol­ecules inter­act through π–π stacking inter­actions [centroid–centroid distances = 3.7457 (14) Å], forming columnar arrangements along the *b* axis. The crystal packing is further enforced by inter­molecular C—H⋯O and C—H⋯N hydrogen bonds.

## Related literature

For general background to organic light-emitting diodes (OLEDs), see: Baldo *et al.* (2000 ▶); Flamigni *et al.* (2007 ▶); Yang *et al.* (2007 ▶); Yersin (2008 ▶). For luminescent Ir^III^ complexes containing 2-phenyl­pyridine or its derivatives, see: Nazeeruddin *et al.* (2003 ▶); Dedeian *et al.* (2007 ▶); Chin *et al.* (2007 ▶); Shen *et al.* (2011 ▶).
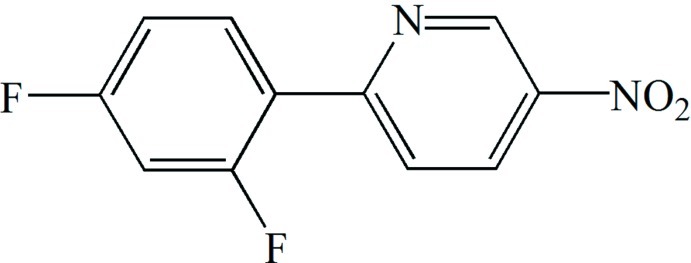


## Experimental

### 

#### Crystal data

C_11_H_6_F_2_N_2_O_2_
*M* *_r_* = 236.18
Orthorhombic, 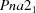

*a* = 22.185 (4) Å
*b* = 3.7457 (6) Å
*c* = 11.894 (2) Å
*V* = 988.4 (3) Å^3^
*Z* = 4
Mo *K*α radiation
μ = 0.14 mm^−1^
*T* = 296 K
0.14 × 0.12 × 0.08 mm

#### Data collection

Bruker APEXII CCD diffractometer
Absorption correction: multi-scan (*SADABS*; Sheldrick, 1996 ▶) *T*_min_ = 0.981, *T*_max_ = 0.989
6331 measured reflections
1750 independent reflections
1450 reflections with *I* > 2σ(*I*)
*R*_int_ = 0.032

#### Refinement

*R*[*F* ^2^ > 2σ(*F* ^2^)] = 0.034
*wR*(*F* ^2^) = 0.081
*S* = 1.06
1750 reflections
155 parameters
1 restraint
H-atom parameters constrained
Δρ_max_ = 0.14 e Å^−3^
Δρ_min_ = −0.12 e Å^−3^

### 

Data collection: *APEX2* (Bruker, 2005 ▶); cell refinement: *SAINT* (Bruker, 2005 ▶); data reduction: *SAINT*; program(s) used to solve structure: *SHELXS97* (Sheldrick, 2008 ▶); program(s) used to refine structure: *SHELXL97* (Sheldrick, 2008 ▶); molecular graphics: *SHELXTL* (Sheldrick, 2008 ▶); software used to prepare material for publication: *SHELXTL*.

## Supplementary Material

Crystal structure: contains datablock(s) I, global. DOI: 10.1107/S1600536812024713/rz2765sup1.cif

Supplementary material file. DOI: 10.1107/S1600536812024713/rz2765Isup2.mol

Structure factors: contains datablock(s) I. DOI: 10.1107/S1600536812024713/rz2765Isup3.hkl

Supplementary material file. DOI: 10.1107/S1600536812024713/rz2765Isup4.cml

Additional supplementary materials:  crystallographic information; 3D view; checkCIF report

## Figures and Tables

**Table 1:** Hydrogen-bond geometry (Å, °)

*D*—H⋯*A*	*D*—H	H⋯*A*	*D*⋯*A*	*D*—H⋯*A*
C10—H10*A*⋯O1^i^	0.93	2.56	3.306 (3)	138
C8—H8*A*⋯N1^ii^	0.93	2.58	3.448 (3)	156
C4—H4*A*⋯F1	0.93	2.40	2.893 (3)	113

Symmetry codes: (i) 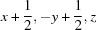; (ii) 
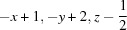
.

**Fig. 1.**
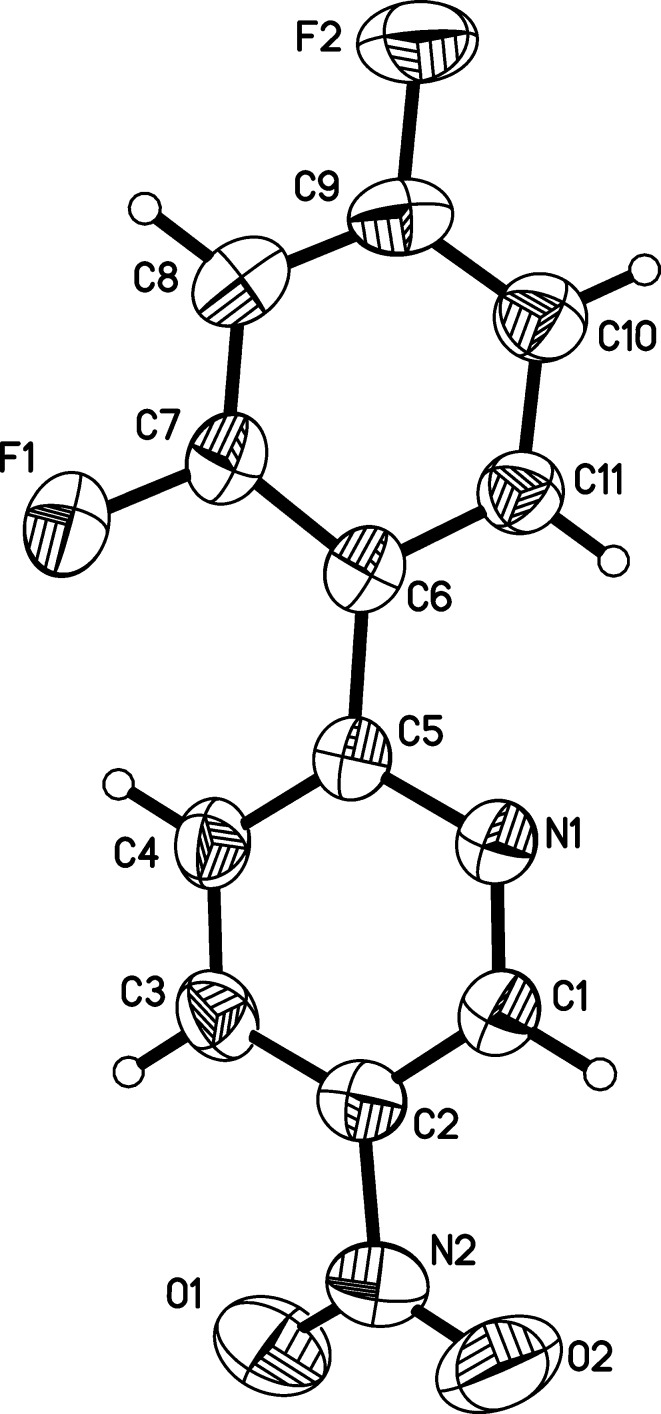
The molecular structure of the title compound, showing 50% probability displacement ellipsoids.

**Fig. 2.**
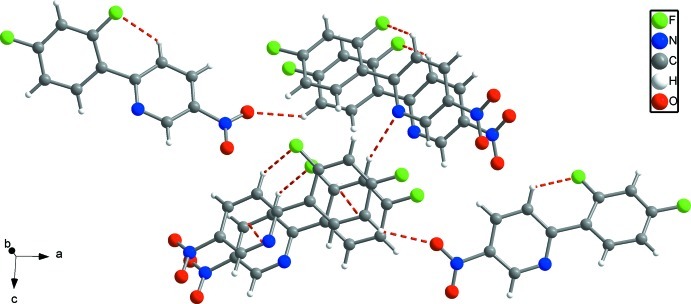
Partial packing diagram of the title compound showing the hydrogen bonding network and π···π interactions as red dashed lines.

## supplementary crystallographic information

### Comment

In recent years, Ir^III^ cyclometalated complexes have received considerable attention because of their outstanding photochemical and photophysical properties, which make this class of complexes widely suitable to a variety of photonic applications and promising emissive materials in organic light-emitting diodes (OLEDs) (Baldo *et al.*, 2000; Flamigni *et al.*, 2007; Yang *et al.*, 2007; Yersin, 2008). Ir^III^ complexes containing 2-phenylpyridine (ppy) and its derivatives are known to exhibit high triplet quantum yields due to mixing the singlet and the triplet excited states *via* spin-orbit coupling, leading to high phosphorescence efficiencies (Nazeeruddin *et al.*, 2003; Dedeian *et al.*, 2007; Chin *et al.*, 2007). It has been concluded that ppy-containing Ir^III^ complexes can emit lights covering a full range of visible colors by introducing electron-donating or -withdrawing groups to the pyridyl or phenyl rings, which can adjust the HOMO-LUMO energy gaps of the complexes (Shen *et al.*, 2011). As a contribution to this research field, we report herein the synthesis and crystal structure of the title compound. The electron-withdrawing fluoro and nitro groups have been introduced on the phenyl and pyridine rings, respectively, of the title compound, and investigations on Ir^III^ complexes containing the title compound will be carried out soon.

The X-ray analysis of the title compound (Fig. 1) shows that the molecule is non-planar, the phenyl and pyridine rings forming a dihedral angle of 32.57 (6)°. The nitro group is slightly skewed with respect to the pyridine ring with a dihedral angle of 12.26 (9)%. An intramolecular C—H···F hydrogen bond (Table 1) stabilizes the molecular conformation. In the crystal structure (Fig. 2), π–π stacking interactions involving overlapping benzene and pyridine rings with centroid-to-centroid distances of 3.7457 (14) Å pack the molecules in columnar arrays running parallel the *b* axis. Furthermore, the columns interact *via* intermolecular C—H···O and C—H···N hydrogen bonds (Table 1).

### Experimental

2-Chloro-5-nitropyridine (3.18 g, 20.0 mmol), 2,4-difluorophenylboric acid (4.00 g, 25.0 mmol) and triphenylphosphine (0.524 g, 2.0 mmol) were dissolved in THF (50 ml). After an aqueous solution of sodium carbonate (2 *M*, 30 ml) and palladium diacetate (0.122 g, 0.5 mmol) were added in, the mixture was refluxed under argon atmosphere for 24 h. After being cooled to room temperature, the reacted mixture was poured into water (50 ml) and was further extracted with dichloromethane (50 ml × 3). The combined extract was washed with saturated brine, dried over magnesium sulfate, and then evaporated to dryness. The crude product was purified by silica gel column chromatography (eluant: petroleum ether/ethyl acetate, 6:1 *v*/*v*), and colourless crystals of the title compound were at last obtained by recrystallization from ethanol in a yield of 70.5% (3.32 g).

### Refinement

All H atoms were positioned geometrically and refined using a riding model with C—H = 0.93 Å for phenyl and pyridyl H–atoms. The *U*_iso_(H) were allowed at 1.2*U*_eq_(C).

### Crystal data

**Table:**

C_11_H_6_F_2_N_2_O_2_	*F*(000) = 480
*M**_r_* = 236.18	*D*_x_ = 1.587 Mg m^−^^3^
Orthorhombic, *P**n**a*2_1_	Mo *K*α radiation, λ = 0.71073 Å
Hall symbol: P 2c -2n	Cell parameters from 24 reflections
*a* = 22.185 (4) Å	θ = 1.9–26.7°
*b* = 3.7457 (6) Å	µ = 0.14 mm^−^^1^
*c* = 11.894 (2) Å	*T* = 296 K
*V* = 988.4 (3) Å^3^	Block, colourless
*Z* = 4	0.14 × 0.12 × 0.08 mm

### Data collection

**Table:**

Bruker APEXII CCD diffractometer	1750 independent reflections
Radiation source: fine-focus sealed tube	1450 reflections with *I* > 2σ(*I*)
Graphite monochromator	*R*_int_ = 0.032
ω scans	θ_max_ = 25.0°, θ_min_ = 1.8°
Absorption correction: multi-scan (*SADABS*; Sheldrick, 1996)	*h* = −24→26
*T*_min_ = 0.981, *T*_max_ = 0.989	*k* = −4→4
6331 measured reflections	*l* = −14→14

### Refinement

**Table:**

Refinement on *F*^2^	Hydrogen site location: inferred from neighbouring sites
Least-squares matrix: full	H-atom parameters constrained
*R*[*F*^2^ > 2σ(*F*^2^)] = 0.034	*w* = 1/[σ^2^(*F*_o_^2^) + (0.0417*P*)^2^] where *P* = (*F*_o_^2^ + 2*F*_c_^2^)/3
*wR*(*F*^2^) = 0.081	(Δ/σ)_max_ < 0.001
*S* = 1.06	Δρ_max_ = 0.14 e Å^−^^3^
1750 reflections	Δρ_min_ = −0.12 e Å^−^^3^
155 parameters	Extinction correction: *SHELXL97* (Sheldrick, 2008), Fc^*^=kFc[1+0.001xFc^2^λ^3^/sin(2θ)]^-1/4^
1 restraint	Extinction coefficient: 0.020 (2)
Primary atom site location: structure-invariant direct methods	Absolute structure: Flack (1983), 823 Friedel pairs
Secondary atom site location: difference Fourier map	Flack parameter: 1.3 (9)

### Special details

**Table:**

Geometry. All e.s.d.'s (except the e.s.d. in the dihedral angle between two l.s. planes) are estimated using the full covariance matrix. The cell e.s.d.'s are taken into account individually in the estimation of e.s.d.'s in distances, angles and torsion angles; correlations between e.s.d.'s in cell parameters are only used when they are defined by crystal symmetry. An approximate (isotropic) treatment of cell e.s.d.'s is used for estimating e.s.d.'s involving l.s. planes.
Refinement. Refinement of *F*^2^ against ALL reflections. The weighted *R*-factor *wR* and goodness of fit *S* are based on *F*^2^, conventional *R*-factors *R* are based on *F*, with *F* set to zero for negative *F*^2^. The threshold expression of *F*^2^ > σ(*F*^2^) is used only for calculating *R*-factors(gt) *etc*. and is not relevant to the choice of reflections for refinement. *R*-factors based on *F*^2^ are statistically about twice as large as those based on *F*, and *R*- factors based on ALL data will be even larger.

### Fractional atomic coordinates and isotropic or equivalent isotropic displacement parameters (Å^2^)

**Table:**

	*x*	*y*	*z*	*U*_iso_*/*U*_eq_	
F1	0.43717 (7)	0.8814 (4)	0.19399 (11)	0.0706 (5)	
F2	0.64096 (6)	0.8761 (5)	0.29027 (14)	0.0783 (5)	
N1	0.39001 (8)	0.6832 (5)	0.52314 (14)	0.0468 (5)	
N2	0.23174 (9)	0.3925 (7)	0.5480 (2)	0.0604 (6)	
C1	0.33559 (10)	0.6124 (6)	0.56308 (18)	0.0487 (6)	
H1A	0.3278	0.6546	0.6388	0.058*	
C2	0.29008 (9)	0.4788 (6)	0.4966 (2)	0.0461 (5)	
C3	0.29993 (10)	0.4202 (6)	0.38367 (19)	0.0499 (6)	
H3A	0.2696	0.3316	0.3376	0.060*	
C4	0.35623 (9)	0.4973 (6)	0.34139 (19)	0.0480 (6)	
H4A	0.3644	0.4647	0.2654	0.058*	
C5	0.40050 (9)	0.6236 (5)	0.41293 (17)	0.0392 (5)	
C6	0.46353 (9)	0.6929 (5)	0.37757 (17)	0.0406 (5)	
C7	0.48022 (10)	0.8139 (6)	0.27144 (19)	0.0457 (6)	
C8	0.53882 (12)	0.8781 (6)	0.2407 (2)	0.0529 (6)	
H8A	0.5485	0.9618	0.1693	0.064*	
C9	0.58240 (10)	0.8137 (6)	0.3194 (2)	0.0524 (6)	
C10	0.57000 (11)	0.6967 (7)	0.4255 (2)	0.0552 (7)	
H10A	0.6007	0.6581	0.4773	0.066*	
C11	0.51028 (9)	0.6372 (6)	0.45341 (19)	0.0468 (6)	
H11A	0.5011	0.5571	0.5254	0.056*	
O1	0.19644 (9)	0.2185 (6)	0.49287 (19)	0.0910 (7)	
O2	0.22205 (9)	0.4973 (7)	0.6427 (2)	0.1016 (8)	

### Atomic displacement parameters (Å^2^)

**Table:**

	*U*^11^	*U*^22^	*U*^33^	*U*^12^	*U*^13^	*U*^23^
F1	0.0672 (10)	0.0979 (13)	0.0468 (8)	0.0059 (9)	−0.0017 (7)	0.0135 (8)
F2	0.0529 (8)	0.1004 (11)	0.0818 (11)	−0.0123 (8)	0.0206 (8)	−0.0020 (9)
N1	0.0453 (11)	0.0560 (12)	0.0390 (11)	−0.0035 (9)	0.0008 (8)	−0.0029 (9)
N2	0.0466 (13)	0.0676 (13)	0.0671 (16)	−0.0070 (11)	0.0044 (12)	0.0016 (11)
C1	0.0491 (13)	0.0584 (14)	0.0387 (13)	−0.0033 (12)	0.0016 (10)	−0.0016 (10)
C2	0.0423 (12)	0.0445 (12)	0.0514 (15)	0.0006 (10)	0.0018 (11)	0.0024 (11)
C3	0.0468 (13)	0.0535 (13)	0.0494 (14)	−0.0034 (11)	−0.0103 (11)	−0.0086 (12)
C4	0.0531 (14)	0.0548 (14)	0.0360 (12)	0.0004 (11)	−0.0040 (11)	−0.0040 (11)
C5	0.0446 (12)	0.0343 (11)	0.0386 (11)	0.0014 (10)	−0.0010 (9)	0.0006 (9)
C6	0.0492 (14)	0.0339 (11)	0.0387 (12)	0.0036 (9)	0.0022 (11)	−0.0021 (10)
C7	0.0543 (15)	0.0437 (13)	0.0392 (12)	0.0042 (11)	0.0012 (11)	0.0027 (11)
C8	0.0614 (16)	0.0494 (16)	0.0480 (13)	−0.0010 (12)	0.0133 (12)	0.0017 (11)
C9	0.0449 (14)	0.0506 (14)	0.0617 (16)	−0.0035 (11)	0.0146 (13)	−0.0053 (12)
C10	0.0486 (15)	0.0605 (16)	0.0564 (16)	0.0036 (11)	0.0012 (12)	0.0028 (13)
C11	0.0443 (13)	0.0487 (14)	0.0474 (13)	0.0022 (10)	0.0016 (11)	0.0038 (11)
O1	0.0561 (11)	0.1191 (18)	0.0978 (18)	−0.0343 (12)	−0.0064 (12)	−0.0072 (13)
O2	0.0777 (15)	0.151 (2)	0.0764 (15)	−0.0335 (14)	0.0293 (12)	−0.0233 (16)

### Geometric parameters (Å, º)

**Table:**

F1—C7	1.351 (3)	C4—C5	1.383 (3)
F2—C9	1.365 (2)	C4—H4A	0.9300
N1—C1	1.324 (3)	C5—C6	1.483 (3)
N1—C5	1.350 (3)	C6—C11	1.390 (3)
N2—O1	1.212 (3)	C6—C7	1.391 (3)
N2—O2	1.213 (3)	C7—C8	1.372 (3)
N2—C2	1.467 (3)	C8—C9	1.367 (4)
C1—C2	1.377 (3)	C8—H8A	0.9300
C1—H1A	0.9300	C9—C10	1.364 (4)
C2—C3	1.378 (3)	C10—C11	1.384 (3)
C3—C4	1.377 (3)	C10—H10A	0.9300
C3—H3A	0.9300	C11—H11A	0.9300
			
C1—N1—C5	118.19 (19)	C11—C6—C7	116.04 (19)
O1—N2—O2	124.2 (2)	C11—C6—C5	119.54 (19)
O1—N2—C2	117.6 (2)	C7—C6—C5	124.4 (2)
O2—N2—C2	118.2 (2)	F1—C7—C8	117.1 (2)
N1—C1—C2	122.4 (2)	F1—C7—C6	119.44 (19)
N1—C1—H1A	118.8	C8—C7—C6	123.5 (2)
C2—C1—H1A	118.8	C9—C8—C7	117.1 (2)
C1—C2—C3	120.1 (2)	C9—C8—H8A	121.4
C1—C2—N2	119.2 (2)	C7—C8—H8A	121.4
C3—C2—N2	120.7 (2)	C10—C9—F2	118.8 (2)
C4—C3—C2	117.8 (2)	C10—C9—C8	123.2 (2)
C4—C3—H3A	121.1	F2—C9—C8	118.0 (2)
C2—C3—H3A	121.1	C9—C10—C11	117.8 (2)
C3—C4—C5	119.4 (2)	C9—C10—H10A	121.1
C3—C4—H4A	120.3	C11—C10—H10A	121.1
C5—C4—H4A	120.3	C10—C11—C6	122.3 (2)
N1—C5—C4	122.1 (2)	C10—C11—H11A	118.9
N1—C5—C6	114.14 (19)	C6—C11—H11A	118.9
C4—C5—C6	123.72 (19)		

### Hydrogen-bond geometry (Å, º)

**Table:**

*D*—H···*A*	*D*—H	H···*A*	*D*···*A*	*D*—H···*A*
C10—H10*A*···O1^i^	0.93	2.56	3.306 (3)	138
C8—H8*A*···N1^ii^	0.93	2.58	3.448 (3)	156
C4—H4*A*···F1	0.93	2.40	2.893 (3)	113

Symmetry codes: (i) *x*+1/2, −*y*+1/2, *z*; (ii) −*x*+1, −*y*+2, *z*−1/2.
